# Evidence of a Causal Role for mid-Ventrolateral Prefrontal Cortex Based Functional Networks in Retrieving High-Fidelity Memory

**DOI:** 10.1038/s41598-018-33164-w

**Published:** 2018-10-05

**Authors:** Peter E. Wais, Olivia Montgomery, Craig E. L. Stark, Adam Gazzaley

**Affiliations:** 1 Department of Neurology & Neuroscape, University of California, San Francisco, USA; 2 Center for the Neurobiology of Learning and Memory & Department of Neurobiology and Behavior, University of California, Irvine, USA; 3 Departments of Physiology and Psychiatry, University of California, San Francisco, USA

## Abstract

Functional neuroimaging studies have implicated regions of both ventrolateral prefrontal cortex (VLPFC) and angular gyrus in processes associated with retrieving goal-relevant information, which increases the fidelity and richness of long-term memory (LTM). To further investigate the roles of these cortical regions as nodes in functional networks with memory regions of the medial temporal lobe (MTL), we used fMRI-guided, 1 Hz repetitive transcranial magnetic stimulation (rTMS) to perturb normal neuronal function. The aim was to test the causal roles of left mid-VLPFC and left angular gyrus (AG) in MTL-VLPFC-parietal networks that have been associated with high-fidelity memory retrieval. rTMS treatments were administered immediately before blocks in an old/new recognition test, which was based on a mnemonic similarity task requiring discrimination of previously studied pictures of common objects. Capability for mnemonic discrimination was evaluated after each of three conditions: placebo control (rTMS at somatosensory cortex), mid-VLPFC target (rTMS at left pars triangularis) and parietal target (rTMS at left AG). The results showed the effect of rTMS perturbation of mid-VLPFC diminished subsequent discrimination-based memory performance, relative to placebo control, and no significant effect of perturbation of AG. These findings show a causal role for functional networks with left mid-VLPFC in high-fidelity retrieval.

## Introduction

Contributions of cognitive control toward the fidelity and richness of long-term memory (LTM) retrieval have been revealed in functional neuroimaging studies that implicate regions of both ventrolateral prefrontal cortex (VLPFC)^1,2^ and the angular gyrus region of posterior parietal cortex (PPC)^3–5^. Consistent with these findings about the anatomical loci of processes associated with retrieval of specific, detailed information, we previously showed that high-fidelity memory is associated with increased functional connectivity of networks between the medial temporal lobe (MTL), VLPFC and PPC^6^. High-fidelity retrieval is based on discrimination of specific details held in memory (i.e., distinctions between target images and related lures).

Although fMRI evidence associated with MTL-lateral cortical memory networks has been replicated, and therefore appears compelling, causal evidence of the functional roles fulfilled by the different network nodes remains elusive. One open question is whether top-down control processes associated with VLPFC, which have been implicated in selection of goal-relevant information from memory^1,2,6,7^, are necessary for successful high-fidelity retrieval? Another question is whether top-down processes associated with angular gyrus (AG) regions of PPC serve a necessary role in retrieval of high-fidelity memories? Based on fMRI results, one prominent interpretation holds that AG function is integral to episodic retrieval^3,4^, while alternative interpretations suggest this region plays a role as a buffer to maintain awareness of different qualities of mnemonic information that might be accessed by other cognitive processes^8–10^. Most recently, AG activity has been positively correlated with the precision of recollection for spatial cues^11,12^, and, interestingly, these findings seem to comport with both earlier models for AG function in LTM.

The motivation here was to address questions about causality of the cortical nodes in MTL-VLPFC-PPC networks that have been associated with LTM based on discrimination of specific details (i.e., high-fidelity information). To accomplish this, we used repetitive transcranial magnetic stimulation (rTMS) to cause transient disruption of normal neuronal function in fMRI-guided network target nodes that are associated with discrimination-based memory^6,13,14^. Immediately preceding memory test runs, 1Hz-rTMS stimulation was applied over target ROIs in left mid-VLPFC (i.e., approximately pars triangularis region we denote with the label PFC), in left AG (i.e., a region we denote with the label AG), and right post-central gyrus as a placebo control (i.e., a region we denote with the label S1). Then, capability for high-fidelity memory retrieval was assessed via performance on a mnemonic similarity task, which required the discrimination of highly similar lures from the actual targets in an old/new recognition test^15^.

Mnemonic discrimination was assessed after each of three active treatment conditions, and behavioral performance was compared between the three conditions to examine whether memory test responses to targets and/or to lures differed on the basis of condition. Support for our hypothesis of a causal role for the left mid-VLPFC and/or the left AG in high-fidelity retrieval would be diminished mnemonic discrimination of highly similar lures after rTMS at those targets, relative to placebo control. We did not expect that rTMS perturbation of lateral cortical regions would have an effect on recognition of the targets because such generalized retrieval does not necessarily depend on discrimination-based memory.

## Results

### Experiment overview

**Figure 1:**
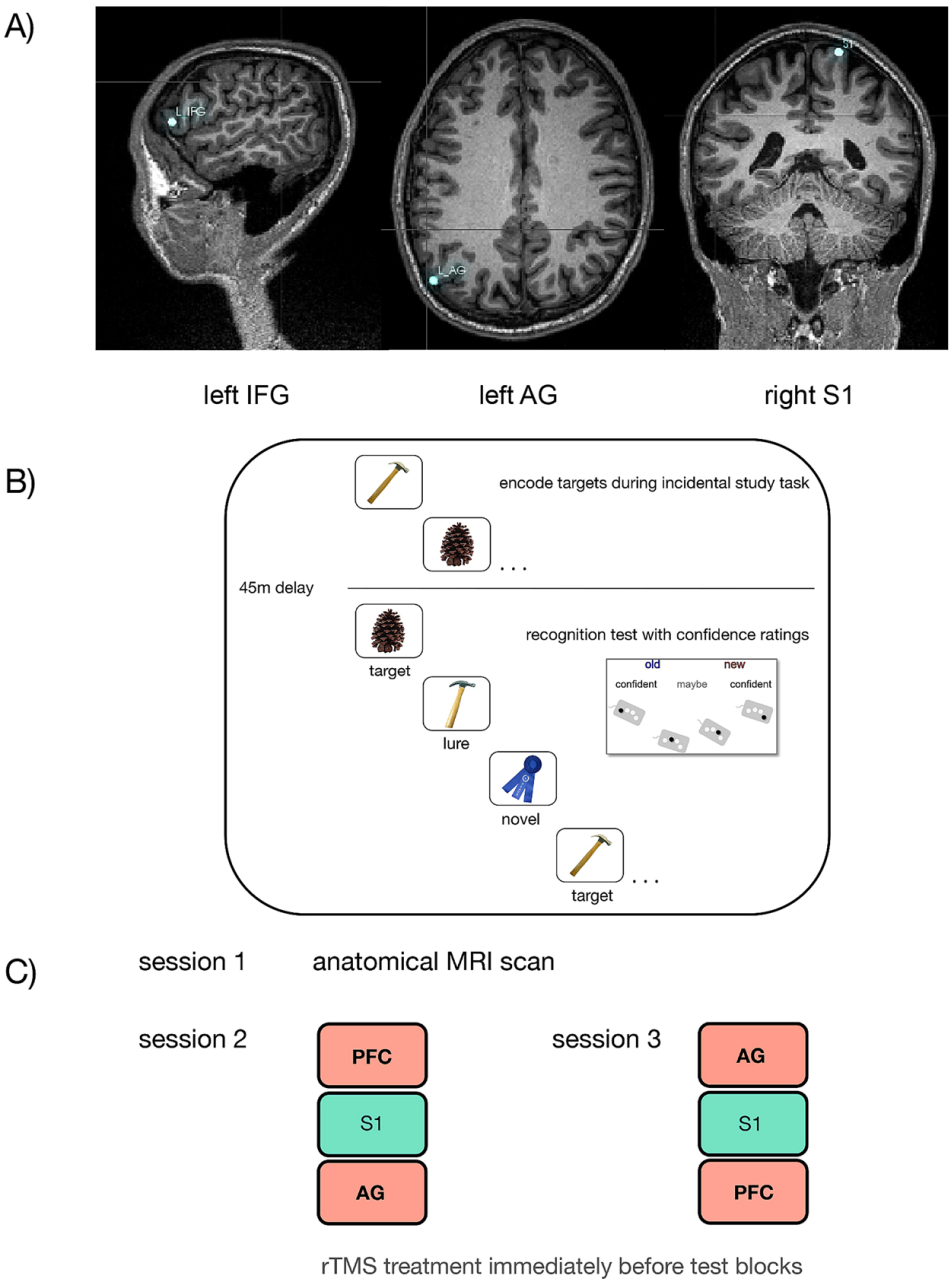
Experimental Procedure. (**A**) A structural scan was collected before a participant (n = 24) completed two test sessions. As illustrated, rTMS treatments were targeted according to fMRI-guided ROI’s (i.e., the left IFG, left AG and right S1) mapped onto each participant’s structural scan using Brainsight™ visualization of their brain in native space. (**B**) The memory test used a mnemonic discrimination task, which included a study session for all target images presented in that session, and then three test blocks each presenting a random order of targets, similar lures and novel images. On each trial, participants gave an old/new response with a level of confidence. (**C**) 1 Hz rTMS treatment was applied for the 8m50s immediately preceding test blocks in the PFC (left IFG target), AG (left angular gyrus target) and S1 (right post-central gyrus) conditions. The two test sessions were spaced approximately two weeks apart.

The experiment was separated into three sessions (Fig. 1): an MRI structural scan, the first rTMS session, and, approximately two weeks later, a second rTMS session. Two rTMS sessions, which presented the exact same treatment/test conditions, were required for each participant in order to balance the randomization order of treatment conditions and collect enough observations for sufficient power in our analyses while complying with our safety protocol’s limitation on the maximum number of rTMS pulses absorbed by a participant in a single day.

Each rTMS session included a study phase for memory encoding and then, after approximately 30 minutes delay, a test phase of memory retrieval with four blocks of an old/new recognition task. Each test block was immediately preceded by one of three different active rTMS conditions (i.e., S1, AG and PFC target sites), or no treatment in the baseline condition. The baseline condition was always the first or last block in each session, and those data are not reported here because the S1 condition served as a placebo control in the analyses.

### Behavioral summary

Performance on the memory tests was assessed via accuracy for old/new responses given the three stimulus categories: Targets (i.e., object stimuli presented during the study sessions), Lures (i.e., the paired object stimuli presented only during the test sessions), and Novels (i.e., completely novel object stimuli presented only during the test sessions). Table 1 shows the group mean proportions for each type of trial response, the mean confidence ratings associated with those responses, and the group mean proportions for each type of response in each of the three conditions.

**Table 1:** Summary of behavioral results.

		Targets				Lures				Novels			
		*hit*		*miss*		*FA*		*CR*		*FA*		*CR*	
proportion		0.84	±0.02	0.16	±0.02	0.28	±0.03	0.72	±0.03	0.04	±0.02	0.96	±0.03
confidence rating		1.85	±0.03	1.55	±0.05	1.67	±0.04	1.85	±0.03	1.03	±0.19	1.97	±0.02
condition:	S1	0.82	±0.02	0.18	±0.02	0.26	±0.03	0.74	±0.03	0.03	±0.03	0.97	±0.03
	AG	0.84	±0.03	0.16	±0.03	0.29	±0.04	0.71	±0.04	0.03	±0.03	0.97	±0.03
	IFG	0.83	±0.03	0.17	±0.03	0.33	±0.05	0.67	±0.05	0.08	±0.08	0.92	±0.05

Mean values are shown for responses during the memory test (SEM). Proportion values were used in the analysis of accuracy, and for Lures and Novels, *FA* = false alarms and *CR* = correct rejections. In the analysis of confidence ratings, values were used such that 2 = definitely and 1 = maybe. Additionally, mean values are listed for each condition (i.e., S1, AG and PFC).

Because the data were collected over two sessions in order to better counter-balance condition order across participants and increase power in the analysis, we compared performance by block order (i.e., first, second and third blocks in each test session) and between sessions. Results showed no effect of block order during each session on mean hit rate (*F*2,46 = 0.380, *p* = 0.69) or mean LureCR rates (*F*2,46 = 2.334, *p* = 0.14). Performance was very similar in the two sessions in terms of both mean hit rate (*F*1,23 = 0.094, *p* = 0.76) and mean LureCR rate (*F*1,23 = 0.223, *p* = 0.64). The principal analysis, therefore, collapsed data from the two sessions by condition and considered differences in performance between test conditions.

### Principal analysis

The effects of rTMS perturbation were examined for recognition of the targets (i.e., hit rates) and discrimination of Lures (i.e., LureCR rates). Repeated-measures 2 × 3 ANOVA compared performance in response categories (hits|LureCRs) and conditions (S1:AG:PFC). The results showed a main effect on response categories (*F*1,23 = 10.049, *p* = 0.004), no effect on conditions (*F*2,46 = 1.159, *p* = 0.322), and an interaction of categories and conditions (*F*2,46 = 3.976, *p* = 0.025). Pairwise comparisons planned in the ANOVA revealed performance differences underlying the interaction with conditions.

**Figure 2:**
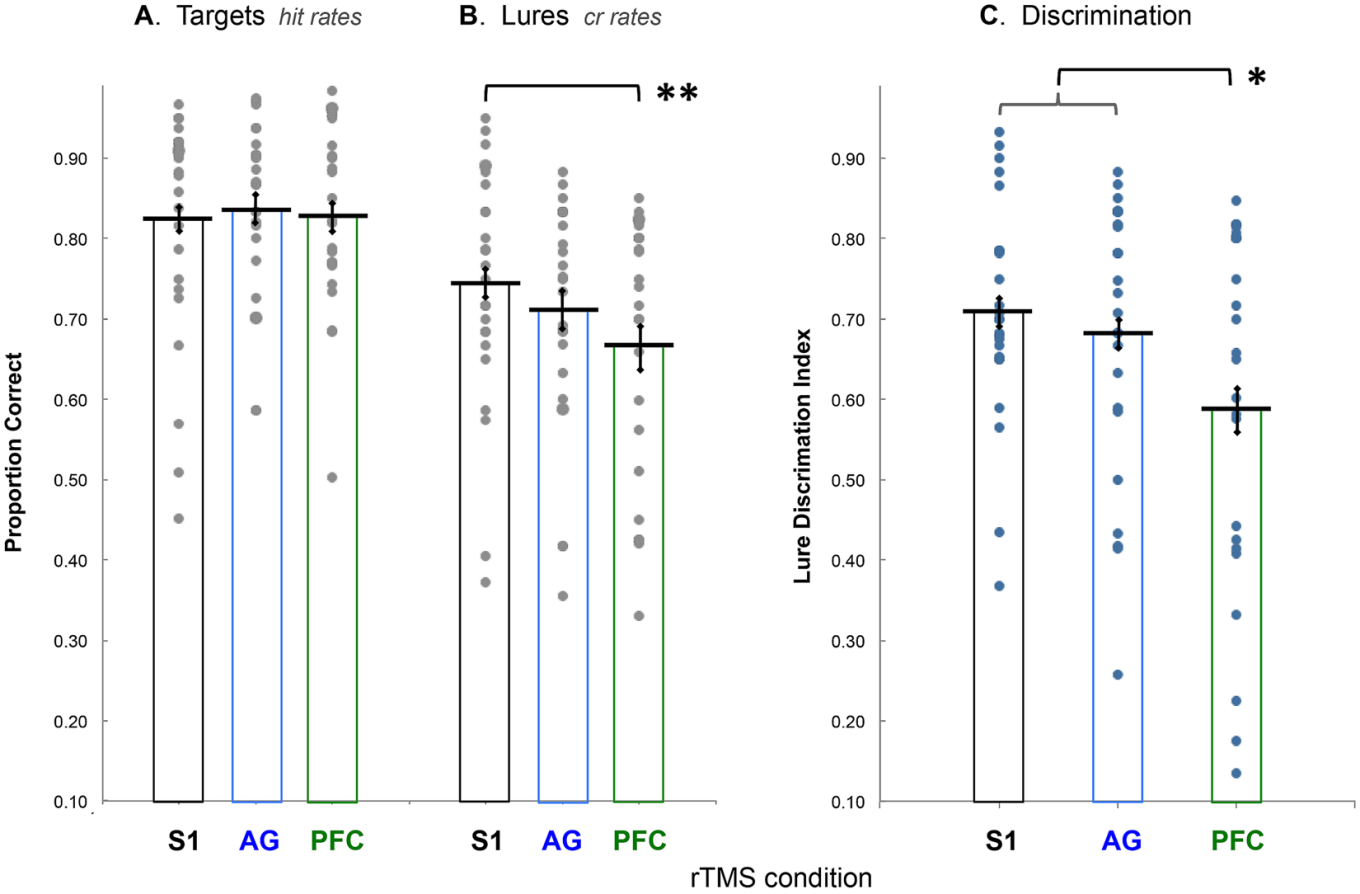
Memory performance by condition. The principal analysis assessed participants’ performance in the memory test in terms of recognizing previously studied targets (**A**) and correctly rejecting similar lures (**B**). Gray dots represent scores by response category and condition for each participant, with black bars indicating group means (SEM). Results were compared, as repeated measures, between two response categories (hits|LureCrs) and three rTMS treatment conditions (S1|AG|PFC). ANOVA revealed an interaction of response categories and conditions such that mean hit rates did not differ by condition, while LureCR rates declined in PFC relative to the two other conditions. Planned pairwise tests showed that mean LureCR decreased following rTMS treatment in the PFC condition, relative to S1, the placebo control condition. Mean LureCR following rTMS treatment in the AG condition was not different than either PFC or S1. **Indicates a difference between conditions, *p* < 0.01. (**C**) Behavioral results were assessed for each participant’s mnemonic discrimination performance using a lure discrimination index such that LDI = proportion LureCR - proportion NovelFA. ANOVA compared LDI results by condition (S1|AG|PFC). The analysis revealed a main effect of condition, and planned pairwise tests showed that performance in the PFC condition decreased relative to S1 and AG. LDI performance in the AG condition was not different than S1. Blue dots show each participant’s mean LDI scores by condition, black bars indicate group means by condition (SEM), and *Indicates a difference between conditions, *p* < 0.05.

Mean hit rates were not different across conditions (Fig. 2A; S1 = 0.82 ± 02, AG = 0.84 ± 03, and PFC = 0.83 ± 03). Mean LureCR rates differed by condition (Fig. 2B; S1 = 0.74 ± 03, AG = 0.71 ± 04, and PFC = 0.67 ± 05), such that correct rejections declined in the PFC condition, relative to S1 (*t23* = 2.86, p = 0.009). Correct rejection rates in the AG condition were not different than in the S1 (*t23* = 1.14, p = 0.267) or the PFC (*t23* = 1.38, p = 0.181) conditions. The pattern in these results shows that perturbation of left mid-VLPFC diminished discrimination of similar lures, and therefore capability for high-fidelity memory retrieval, relative to the placebo control.

The absence of a main effect of condition in the principal analysis was notable because it indicated that effects of rTMS, on average, did not shift participants’ criterion to respond “old” across test blocks. For example, if results in the PFC condition showing increased false alarms, relative to other conditions, reflected simply the shift to a more liberal criterion to respond “old,” then the mean hit rate would have also increased in that condition. The mean hit rate was unchanged in the PFC condition, however, while the mean LureFA increased, as revealed by the condition X response interaction.

### Confidence ratings analysis

Comparisons within the stimulus categories showed the typical pattern that confidence tracks accuracy. The group mean rating associated with hits was greater than with misses *(t23* = 7.72, p < 0.001), with LureCR greater than with LureFA *(t23* = 4.86, p < 0.001), and with NovelCR greater than with NovelFA *(t23* = 4.79, p < 0.001).

Following the approach in the principal analysis for response accuracy, a *post hoc* analysis used repeated-measures 2 × 3 ANOVA to compare confidence associated with correct or incorrect responses (hits|LureCRs, or misses|LureFAs) by condition (S1:AG:PFC). Results for correct responses (i.e., the hits and lure correct rejections) showed no effect of condition (*F*2,46 = 0.177, *p* = 0.838) and no interaction of response category and condition (*F*2,46 = 0.119, *p* = 0.889). Similarly, results for incorrect responses (i.e., the misses and lure false alarms) showed no effect of condition (*F*2,46 = 1.099, *p* = 0.342) and no interaction of response category and condition (*F*2,46 = 0.92, *p* = 0.406).

### Follow-up analysis

The randomization of targets and lures in each test block and counter-balancing of blocks across conditions and sessions caused one-half of the targets to be presented “first,” before paired lures in each session, while one-half of the lures were presented “first,” before paired targets. Because “first” presentations might have cued responses for the paired targets or lures in second presentations, we examined how accuracies for the first presentations compared to overall results by condition from the principal analysis above. ANOVA comparing two presentation orders X three conditions in target accuracies showed a main effect of order (*F*1,23 = 6.844, *p* = 0.012), such that hit rates were higher for targets presented “first.” As we expected, there was no main effect of condition or interaction of order with condition. ANOVA comparing accuracies for lures showed a main effect of condition (*F*2,46 = 8.045, *p* = 0.006), such that correct rejection rates were lower in the PFC than S1 or AG conditions. Unlike the pattern for targets, accuracy for lures was not different between those presented before or presented after paired targets (*F*1,23 = 2.361, *p* = 0.131).

### Lure discrimination index

An additional assessment of participants’ mnemonic discrimination capability is possible using a Lure Discrimination Index (i.e., LDI = proportion LureCR – proportion NovelFA) to correct for noise in memory responses^6^ (and see 3AFC)^16^, such as guessing that might vary between rTMS conditions. Because our experimental procedure was restricted on the total rTMS pulses allowed per session by participant safety protocol, we optimized power for the principal analysis with total observations for paired targets and lures. As a consequence, timing was available for only a small number of novel stimuli per test block. Notwithstanding the possibility of quantization error around mean NovelFA for the limited number of those observations, we followed up the principal analysis with ANOVA comparing LDI results by condition (S1|AG|PFC). Results showed a main effect of condition (*F*2,46 = 5.526, *p* = 0.009) (Fig. 2C), and planned pairwise comparisons revealed that performance in the PFC condition was reduced relative to S1 *(t23* = 2.78, p = 0.011) and AG *(t23* = 2.66, p = 0.014), and the AG condition was not different than S1 *(t23* = 0.93, p = 0.365).

Notably, identical encoding and test instructions were read to participants in sessions one and two. Because participants may have anticipated the encoding procedure during session two differently than the same incidental encoding instructions for session one, a follow-up analysis compared LDI performance in each condition (i.e., rTMS ROI) between sessions one and two. The results confirmed that LDI performance by condition did not differ between sessions (S1 1 vs. 2, *t23* = 1.33, p = 0.197; AG 1 vs. 2, *t23* = 0.34, p = 0.737; PFC 1 vs. 2, *t23* = 0.42, p = 0.678), which indicate that greater familiarity with the procedure during session two did not affect rTMS effects by condition.

## Discussion

Functional neuroimaging studies recently described distributed MTL-cortical networks that are engaged during retrieval of high-fidelity memory. These functional connectivity results were associated with LTM for detailed, but not generalized, memories^6^, with retrieval of specific features of rich auditory memories^2^, and with superior free recall after six weeks of cognitive training^17^. The implication from these correlative findings is that high-fidelity LTM arises from increased, network-coordinated activity between the MTL, a region shown to be necessary for LTM^18,19^, and several cortical regions associated with cognitive control^1^ and mental imagery for the specific features of memoranda^3^.

The aim in our study was to probe the causal role of two distinct high-fidelity memory networks, which were recently identified in an fMRI connectivity analysis^6^. The results here showed that discrimination-based memory performance declined, relative to a placebo control condition, immediately after normal neuronal activity in the PFC network node was disrupted with 1 Hz rTMS. This is the first causal evidence, so far as we are aware, linking contributions from VLPFC regions and their functional networks to successful goal-directed retrieval of detailed information from LTM. Specifically, perturbation of left mid-VLPFC, relative to perturbation of S1 or a left angular gyrus region, resulted in diminished LDI performance. These outcomes inform interpretation of the necessity of these nodes in functional MTL-cortical networks that drive high-fidelity retrieval. We discuss the current understanding about LTM retrieval processes associated with MTL-VLPFC and MTL-AG networks, and then the implications of the behavioral findings and causal evidence reported here.

Neuroimaging studies have found that functional MTL-cortical networks are associated with processes supporting mnemonic selection, awareness of task-relevant details and reconstruction of visual details^1–4,6,20^. Specifically, increased activity and functional connectivity along an anterior-posterior gradient in VLPFC have been associated with control processes contributing to retrieval of detailed memories^21,22^. These different subregions of VLPFC share reciprocal connectivity^21^. Although all of these processes may contribute in concert toward discrimination-based judgments that underlie high-fidelity retrieval, one report makes a dissociation clear^1^. Processes associated with anterior VLPFC (i.e., pars orbitalis, approximately BA47) were shown to control retrieval of specific episodic information, relative to processes associated with mid-VLPFC (i.e., pars triangularis, approximately BA45) shown to control selection of contextual or semantic information from competing or overlapping memories. Notably, this dissociation in the functional organization of controlled retrieval processes associated in fMRI results with different VLFPC subregions is convergent with results from patients with focal prefrontal cortex lesions^7^.

For the current results, diminished discrimination performance caused by perturbation with rTMS was manifest as increased mean false alarm rates to similar lures and novels after treatment of the mid-VLPFC target (i.e., PFC condition), relative to the S1 placebo control (Table 1). Importantly, mean hit rates did not differ between the three conditions in our study. This pattern of results shows that disruption in the PFC condition diminished the capability to correctly reject the similar lures, and hence increase false alarms, yet did not cause a shift to increase “old” responses to the targets. Taken together, these data indicate that discrimination of details held in memory was impaired in the PFC condition, but general bias to respond “old” to stimuli was not.

Indeed, the outcome showing false memory errors increased in the PFC condition provides direct evidence that left mid-VLPFC function is necessary in networks engaged during successful mnemonic discrimination. Additionally, our finding for this causal role is convergent with results showing impairment in contextual memory among patients with structural MRI evidence of mid-VLPFC lesions^7^. Neuroimaging findings have associated increased functional connectivity between mid-VLPFC and MTL with successful resolution of interference between similar or overlapping representations held in mental imagery^6,23–25^, and we propose that these networks facilitate accurate tuning of high-fidelity memory.

rTMS perturbation of AG had no effect on either mean hit rate or mean false alarm rate, relative to S1. LDI results showed a numerical reduction in performance in the AG condition, based on decline in discrimination for the narrow majority of participants, but not a significant decline as found in the broader effects in the PFC condition. Our results for the AG condition, therefore, do not add significant new information to the emerging literature focused on the functional necessity of AG in LTM retrieval^11,12,26–29^.

The current literature relevant to AG function includes several data points, along with their associated interpretations. The neuroimaging literature that has examined lateral parietal function in LTM, including our own report^6^, offers support for AG involvement in high-fidelity retrieval, based on the interpretation this region acts as a flexible buffer engaged to represent episodic and associative details encoded in upstream association cortex areas^3,27^. Additionally, fMRI results show that reactivation of encoded category information and visual imagery for episode-specific details is associated with robust patterns of AG activity^4^. Yet, relevant patient studies belie the functional necessity of PPC for accurate source recollection and instead suggest its role may be in the experience of confidence about episodic retrieval^28^.

Two recent studies testing the causal role of AG have added important support for its functional necessity in different qualities of LTM than that examined by our study. Nilakantan and colleagues found improved precision of recollection for spatial information after stimulation of AG function with repeated 20 Hz treatments of rTMS^12^. Thakral and colleagues showed the amount of autobiographical information recalled was diminished after 1 Hz rTMS was targeted to disrupt AG function^29^. What appear to be different conclusions between our results and those from these other rTMS studies, however, are not likely exclusive. There are important differences between the form of memory retrieval we tested (i.e., mnemonic discrimination) and effortful autobiographical recall as tested by Thakral and colleagues^29^, or precision in spatial memory tested by Nilakantan and colleagues^12^. In our example, resolving mnemonic interference or monitoring source memory may be two distinctions in processes supporting episodic retrieval wherein AG does not serve a critical role^24,25,28^. Further research is needed to explore the concept that AG function may be to distribute information available from LTM as needed in service of other higher cognitive demands.

A few limitations in our procedure and rTMS, more generally, suggest caution about our conclusions. The experiments targeted only left hemisphere nodes of the previously identified functional high-fidelity retrieval networks^6^, although both those and other relevant fMRI findings implicated bilateral organizations of memory networks in association with detailed recall and reinstatement of mental imagery from LTM^3,17^. Because safety protocols precluded simultaneous bilateral stimulation in our experiments, we targeted fMRI-guided nodes only in the left hemisphere, which had been associated with the most robust functional network results. Our results, therefore, cannot answer whether simultaneous bilateral PFC stimulation would cause even greater deficits in discrimination-based retrieval, or simultaneous bilateral AG stimulation would cause discrimination deficits for a larger, and significant, proportion of participants.

Finally, results evidencing a causal role for mid-VLPFC in goal-directed retrieval do not preclude a substantial role for anterior VLPFC in high-fidelity LTM, given that interactions between anterior and mid-VLPFC regions typically accompany effortful retrieval^21,30^. An interesting challenge for future research using rTMS of anterior VLPFC (i.e., BA47) will be to demonstrate whether perturbation of that region diminishes recall for episodic details selectively, in contrast to the broader memory effects we report here.

## Materials and Methods

### Participants

Twenty-seven adults were recruited in this study. Inclusion criteria were: native speakers of English, right-hand dominance, completion of 14 or more years of education, normal or corrected-to-normal vision, free of any psychotropic medications and/or conditions contra-indicated for MRI, and family history clear of seizure or epilepsy. Participants gave their informed consent in accordance with the Institutional Review Board of the University of California, San Francisco, and received a small fee in compensation for their time. All experimental methods were carried out in accordance with the guidelines approved for this research under UCSF IRB #15–16824. Three participants’ datasets were excluded from analysis because: one participant did not tolerate stimulation at left PFC during her first session, one participant fell asleep during the baseline test block during his first session, and computer malfunction corrupted data for one participant. Data collected from 24 participants (14 males) between the ages of 18 and 30 years were included in the final analysis.

### MRI structural scan and rTMS target site selection

A high-resolution T1-MPRAGE scan (1 mm isotropic resolution) was collected for each participant using a Siemens 3 T Magnetom Trio and compiled with Analysis for Functional NeuroImages (AFNI_2015) software (Cox & Hyde, 1996). A virtual three-dimensional cortical surface was rendered for each participant using their original space T1 model with Brainsight™ TMS software, version 2.2, © Rogue Research, Inc. Using visualization of each participant’s Brainsight™ model, stimulation targets were located at the native-space homologues of functional ROIs identified by group results in Wais *et al*.^6^. The anatomical precision of rTMS targets for each participant was maintained by inverse-warping the locations derived from the group fMRI results and mapping those locations to each participant’s native-space brain rendering (Fig. 1A). Peak voxels for those functional ROIs were situated on the inferior frontal gyrus (approximately BA45, pars triangularis, x = −46, y = 35, z = 1 in Talairach space) and the angular gyrus (BA39, x = −55, y = −57, z = 34). For the rTMS target sites, PFC and AG were aligned to the central voxels of the respective functional ROIs and the right S1 ROI was mapped onto each participant’s Brainsight™visualization of the post-central gyrus, approximately 5 mm lateral from midline.

### Stimuli

512 object images were organized into two, equivalent sets, so that participants saw unrelated stimuli during each session. Each stimulus was a photograph of a common object, in color and centered on a white screen (viewed on a 15-inch diagonal LCD from a distance of approximately 23 inches), from a subset of the Mnemonic Similarity Task^15^. In each of the two sessions, 120 target objects were paired with 120 very similar lures, and the remaining 16 stimuli were distinctly novel objects. Images were displayed at 768 × 1024 pixel resolution on an LCD computer monitor, using E-prime 2.0 (Psychology Software Tools, Inc.; Pittsburgh, PA).

### Procedure for memory assessment

We presented an incidental task first as a study phase, and after an interval of approximately 30 minutes, an old/new recognition task was used in a test phase (Fig. 1B).

In the study phase, participants viewed one target image on each trial (stimulus presentation = 2.5 s) while responding to incidental questions designed to promote in-depth visualization of each object: 1) “yes or no, will the object fit inside a ladies medium shoe box?”; and 2) “yes or no, can you carry the object across the room using only your right hand?”. The two questions were presented in separate runs so that participants viewed each target image twice.

During the interval between study and test, after an opportunity to stretch, participants were aligned and thresholded for the rTMS treatments. Participants were kept naïve to the memory test until being instructed for their first test block. Once positioned comfortably for rTMS treatment and wearing a headband tracker for the frameless stereotaxic software, participants were instructed about the memory test (for the first experiment session, this constituted a surprise memory test, but repetition of the protocol was obvious to participants during the second experiment session). After a brief practice run of the test procedure, the inter-leaved sequence of rTMS treatments and test blocks began.

Each of the test blocks included 64 trials and was divided into two runs such that participants were cued at the mid-point by on-screen instructions to take a brief break (7 s). 30 targets (i.e., studied objects), 30 lures (i.e., very similar objects) and four novel items (i.e., distinctly new objects) were presented in each block, and the total run time of each block was 5 m 27 s. Presentation of target, lure and novel trials was randomized within each block, and block order was counter-balanced across of participants. A target and its paired similar lure were never presented in the same test block, and the order of target first or similar lure first was counter-balanced across all the trials. Because one-half of the targets were presented at test before their paired lures while one-half of the lures were presented first, we planned an additional comparison, by condition, of accuracies based on first and second presentations to answer whether either version had effects on the date (see follow-up analyses in Results).

The trial procedure presented one object image (2 s) and then a response screen (2 s) that cued the participant to enter an old/new recognition rating on a response box held in the right hand according to a four-level confidence scale. Participants were instructed to respond whether each item was 1 = definitely old, 2 = maybe old, 3 = maybe new, or 4 = definitely new. Fixation (1 s) separated each trial. Considering the 30-minute break between study and test phases, and the average duration and counter-balanced order of conditions in the test phase, the approximate session retention interval was 54 minutes running from the mid-point of the study phase to the mid-point of the test phase.

For analysis, trials were later sorted into hits (“old” responses to targets), misses (“new” responses to targets), lure correct rejections (LureCR: “new” responses to lures), lure false alarms (LureFA: “old” responses to lures), novel correct rejections (NovelCR: “new” responses to novel items), and novel false alarms (NovelFA: “old” responses to novel items) on the basis of participants’ responses.

### Mnemonic discrimination task

Our study utilized a behavioral paradigm that provides both a traditional measure of explicit recognition, as well as measures to assess successful mnemonic discrimination. Discrimination requires memory of the details that make an episode unique, while generalization results from an overall match with information stored in LTM. Recognition of a target as “old” can be based on contributions from both processes. Correct rejections of the lures, however, indicate memory judgments based on discrimination of their differences from targets, which likely involves specific details relevant to the studied target. In contrast, false alarms to similar lures indicate generalization-based memory (i.e., old/new recognition based on low-fidelity memory), or possibly guesses about old/new decisions on trials when no memory for the type of object presented was retrieved. Consequently, we characterized memory retrieval based on discrimination as having high fidelity and memory based on generalized recognition as having low fidelity^6^.

Our principal analysis of memory performance focused on a behavioral measure of each participant’s discrimination of the Lures as new during the old/new recognition task. Any given correct rejection may have identified differences between a Lure and its paired Target, or between a Lure and all other objects presented in the procedure. In either case, correct rejection of a Lure as “new” indicates a memory judgment driven by underlying discrimination of details highly relevant for the retrieval goal.

### rTMS threshold and alignment

1 Hz magnetic stimulation pulses were applied on the scalp with a Magstim 70 mm figure-of-eight induction coil Standard Rapid TMS Unit. A broad interpretation of the effect caused by rTMS is that it induces an increased amount of neural noise in the cortical target^14^.

Susceptibility to rTMS perturbation is influenced by structural heterogeneity in cortex^31,32^. Stimulation procedures must take into account, therefore, allowances for variability in skull thickness and individual sensitivity in order to achieve consistent dosage across ROI’s and participants^33^. For each participant, we determined the effective rTMS intensity required to achieve perturbation of cortex by observing and recording their left motor cortex excitability threshold (MT)^31^. Each participant’s MT value, therefore, was set as the percentage of maximum stimulator output that produced a reliable motor response, as recorded on the Magstim Standard Rapid controller. The mean MT stimulation intensity for the study cohort was 75% ± 8. The specific stimulation intensity for each target ROI was then recalculated using the approach of Stokes *et al*.^32^ to adjust MT for mean skull thickness at the regional coordinates of each ROI. Accordingly, stimulation intensities were set for each participant as follows: S1 at 100% of MT, AG at 95% of MT, and PFC at 80% of MT. For the study group, therefore, mean stimulation intensity at right S1 was 75 ± 8%, mean stimulation intensity at left PFC was 60 ± 6%, and mean stimulation intensity at left AG was 71 ± 7%.

Preceding the test phase, the position of the participant’s head and a 3D rendering of their cortical surface were co-registered using Brainsight™ frameless stereotaxic software, which enabled precise targeting of the three rTMS target sites. During rTMS treatments, each with a duration of 8m50s, the Magstim coil was hand-held flat against the participant’s scalp by the experimenter, such that its handle was approximately parallel to an estimation of the participant’s Sylvian fissure during the PFC and AG conditions. The experimenter maintained contact at the scalp over the fMRI-guided ROI, and the targeting software interface typically showed treatment was applied at a total deviation of 0.8 mm from ROI coordinates (i.e., x, y and z axes).

### rTMS procedure

The order of the three conditions was counter-balanced and pseudo-randomized across participants on the basis that treatment to the left hemisphere was not applied during two blocks in succession, and the order was different between a participant’s two rTMS sessions. Immediately prior to each test block, 1 Hz rTMS pulses were applied at the scalp for 8m50s over the targeted ROI (Fig. 1C). During treatment periods, participants wore earphones as protection against noise associated with pulses from the rTMS coil.

Participants were strongly encouraged to listen to music tracks of their choosing via earphones during each active treatment period in order to remain alert, and all participants did so. Participants were queried about possible drowsiness during post-experiment debriefing, and all but one confirmed steady alertness across all conditions. Data from the one participant who did report drowsiness was excluded from the analysis.
